# Systematic Use of Music as an Environmental Intervention and Quality of Care in Nursing Homes: A Qualitative Case Study in Norway

**DOI:** 10.3390/medicines6010012

**Published:** 2019-01-18

**Authors:** Kari Bjerke Batt-Rawden, Marit Helene Sund Storlien

**Affiliations:** 1 Department of Health Sciences, Norwegian University of Science and Technology (NTNU), 2815 Gjøvik, Norway; 2 Department of Nursing, Norwegian University of Science and Technology (NTNU), 2815 Gjøvik, Norway; marit.storlien@ntnu.no

**Keywords:** music, singing, intervention, nursing, education, implementation, health, wellbeing, quality of care, innovation

## Abstract

**Background:** The systematic use of music as an environmental intervention in nursing homes shows beneficial effects on patients’ health, safety, and quality of life in a care-related perspective. A county in Norway and a Nursing Education Department in a region of Norway collaborated on the project “systematic use of music as environmental intervention and quality of care in nursing homes” for nursing students. **Methods:** This study from Norway (2017) had a qualitative and explorative approach. The sample (*n* = 33) was strategically and conveniently selected. Seven different focus group interviews consisted of nursing students, practice counselors, teachers, and project leaders, representing three nursing homes and healthcare centers. Passive observation lasting two days in each of the six departments was executed in order to observe environmental treatment in practice. **Results:** The beneficial aspects of using music as an environmental intervention in nursing homes increased among the students, and contributed to improved interaction, communication, and development of care with the patients. Students who participated actively in musical interaction such as improvisation, singing, and music listening with the patients were committed and motivated. The staff and management showed varied enthusiasm for the project. **Conclusions:** If the systematic use of music as environmental therapy and quality of care in elderly care is to be successful, it seems vital to include this theme early in nursing education. By creating early involvement among nurses, it might influence, inspire, and encourage involvement among employees and management.

## 1. Introduction

### 1.1. The Value of Music and Singing for Health and Wellbeing

There seems to be a consensus among professionals that music can influence the body, mind, and spirit. In a sense, this consensus reflects universal principles of life depicted by classical theories of music and medicine; however, its basic assumptions were gradually investigated scientifically and documented carefully [1,2,3,4,5,6,7,8,9]. There are varied methods of accounting for different professional musical interventions that represent the various discourses. Modern western diagnostic practices are often designed to exclude, or at least isolate, spiritual or body/mind aspects of illness in order to focus more clearly on the biological processes [10]. This is opposed to how music therapists or musical healers seem to be aware of the dualistic body/mind approach and transfer this view into their practice [11,12,13,14,15,16,17,18,19,20]. In some societies, there was experimentation in combining indigenous healing practices and modern medicine or music therapy, which was successful and aimed at bringing the best of both worlds to those who are suffering.

There seems to be a growing scientific acknowledgement of music’s ability to elicit physiological, psychological, and cognitive responses and entrainment, and to evoke imagery and associations that seem to be unique for each individual [1,21,22]. In addition, music seems to have an enhancing or diminishing effect when combined with other methods of treatment. Current professionals seem to be gradually extending their boundaries toward interdisciplinary cooperation and collaboration as a way to collect further data, and improve techniques and methods which can build part of an evidence-based knowledge about music’s power in relation to health, healing, and illness in western societies [8,23,24,25,26,27,28,29,30]. One example of this theme is where some authors suggest that nurses should be taught how to incorporate music interventions into their practice to more effectively manage anxiety and individualize patient care [31].

Medical music intervention aims at facilitating the process of treatment, reducing stress, or enhancing well-being, and is basically concerned with pre-recorded music for patients [9,12,32,33,34,35]. Music is widely used as an adjunct to a number of non-medical treatments such as biofeedback, hypnosis, meditation, psychotherapy, exercise, diet, and imagery [21,22,36,37,38,39,40,41,42]. By giving patients a choice of taped music to listen to through headphones [31,43,44,45], music listening seems to be linked with a reduction in stress hormone levels, anxiety, pain, and heart rates, as well as a diminished need for anesthesia, lowered pulse and blood pressure rates, and reduced post-operative pain and need for analgesic medication. Furthermore, music listening seems to improve comfort, disclose emotions, ventilate feelings, enhance self-regulatory procedures and wellness, hasten recovery, and shorten length of stay in a variety of healthcare settings [21,22,34,43,44,45,46,47].

From this perspective, several authors argue how the nursing profession should consider music as a complement to conventional care, stressing the importance of providing personal stereos or headphones designed to reduce psychological and physiological stress associated with surgery. The hospital environment may also appear threatening in the sense that it is necessary to provide an individualized approach to preoperative patient care [21,22,45,46,47]. Music can be an effective intervention, because it serves as a distraction from unpleasant stimuli and helps patients gain a certain control over a strange environment [48,49]. 

Scholars argue that patients ought to listen to their own choice of music, for example, during surgery experience or during pre- or post-treatment and rehabilitation, which ought to affect patients’ postoperative recovery [1,5,31,44,48,49]. Some scholars also suggested how nurses can, for example, establish libraries of music that were shown to improve outcomes for specific patient populations [50]. This notion emphasizes that music interventions in hospitals ought to be viewed as pleasurable and enjoyable social activities [51]. Some scholars also suggested that nurses ought to develop their personal healing qualities, which might increase awareness of healing in their own lives, and that the time has, thus, come to make music interventions an integral part of nursing practice [46,50,51,52]. 

The effectiveness of music therapy as a treatment modality in and of itself continues to grow in importance [22,52,53,54,55,56], particularly strategies and methods that focus on therapy as a means of contact and communication by motivating and creating atmospheres that break isolation. According to Wigram et al. [1], the crucial difference between music healing and music therapy is said to be ontological and involves developing a musical relationship with the patient/client, which encompasses the healing power of music, ideally teaching clients to build up a conscious relationship with themselves by increasing their social skills and competence [1]. 

A holistic view of brain function in relation to music seems to be helpful to describe how individuals may retrieve information through reminiscence work [57,58]. It is of particular benefit for people with forms of dementia such as Alzheimer’s disease, where severe frustration and depression can result from deterioration in cognitive and verbal functioning [33,58]. The current standards in geriatric care recommend non-pharmacological approaches to these challenges, including safe approaches to managing pain and stress, enhancing symptom relief, and fostering independent lifestyles with the highest quality of life possible [59,60]. In work with the elderly, people may often ask for songs to remember happy or sad events in their life, allowing tears of sadness or happy memories to emerge while working through their feelings of loss [23]. Music-based environmental therapy has a positive effect on wellbeing and drug consumption, and music has a particularly good effect in the face of unease and depression in patients with dementia [1,58,60,61,62,63,64,65,66]. 

There is a well-developed base of knowledge on how singing may promote health for clinical and non-clinical groups [28,67,68,69]. The power of singing as an empowering, enchanting, or enthralling activity should not be underrated, but be seen as a vital health-enhancing aspect of everyday life [70,71]. Singing does result in measurable physiological changes. Research into singing showed widespread effects on the immune system through reduced levels of cortisol, and increased levels of cytokines and salivary immunoglobulin A (IgA). It is associated with altering oxytocin levels and emotional states [72,73]. Physical effects include changes in breathing, posture, and relaxation, as well as heightened alertness, reduced fatigue, and stress relief [74,75,76].

### 1.2. Theoretical and Conceptual Framework

In this paper, we sought inspiration from sources that serve as a conceptual and theoretical framework for music as a health resource. Firstly, Ruud’s work [6,7,19,20] points to how music may be a “kind of technology” [20] that improves health and contributes to the quality of life, and is, thus, relevant for the promotion of health. Ruud [6] suggests that music gives us a cultural platform from which to make our decisions on matters concerning our life, and discusses how music may satisfy some human needs. He introduces four aspects in which music may contribute to the quality of life. The first aspect concerns music’s ability to experience emotional nuance, to experience and express various degrees of intensity, and to maintain precise concepts of feelings. This perspective may be linked to a sense of having a “real” self, or a self that is felt as natural and “true”, or a self that is felt in accordance with how life is experienced in general. From this viewpoint, music may give a sense of personal relief, vitality, and strength, transcending a subjective feeling of being burdened by physical or mental troubles. Vitality can imply our ways of activating a certain range of feelings, as well as how we integrate our experiences and express them clearly. 

Secondly, music is a tool for developing agency and empowerment. An important aspect of health is related to the ability to take responsibility for one’s life and actions, to be able to make choices, and to follow a plan one has set. When health is the issue, it is observed how one important factor related to our medical culture is the feeling of disempowerment. Through listening to music and engaging in conversation about music with other people, we learn about how other people experience music and life in society in general, thus acquiring a basic social competence.

The third aspect is related to the concept of belonging. The modern way of life sometimes leads to insecurity, breakdown in families, and increased mobility in the population, followed by individual isolation and loneliness. Through the intimate frame given by musical activity, individuals are bound together through common musical experiences. As Ruud [6] argues, being with others may provide intense experiences of involvement, and a heightened feeling of being included; thus, music becomes a social resource.

The fourth aspect describe involvement with music as a way of providing meaning and coherence in life. Ruud [20] builds on the work of Antonovsky [77,78], and argues how the individual’s sense of coherence and meaning in life could contribute to the general resources of resistance toward illness. In the sense that our musical experiences are remembered or felt as being significant, this relates to discussions concerning emotional and bodily involvement in music. From these perspectives, Ruud [6] argues further how the process of music-making allows for musicking (Musicking: To music is to take part, in any capacity, in a musical performance, whether by performing, by listening, by rehearsing, by practicing, by providing material for performance (i.e., composing), or by dancing. [79]. See also Elliott’s definition of musicking: all human action related to music: http://www.davidelliottmusic.com/) as a way of empowering our lives, trying out methods of behavior which affect our social relations, boundaries, and self-reflexivity, thus providing a better quality of life and better health.

DeNora’s work [8,9] on music in everyday life illuminates how music serves as a spectrum of everyday needs and applies the concept of “affordance” to argue how music may allow certain kinds of uses or interpretations, as well as how music is present in a variety of social and personal contexts where mood is regulated, attention focused, or energy channeled. Her work highlights the creative and resourceful ways people use music and, to varying degrees, how people use music to construct themselves in situated and imaginatively situated relation to others. Music may provide resources for the recovery of self-identity, understood as musically afforded opportunities for interaction, for reminiscence, and for the adoption of roles and emotions in, during, and after musical activity. This framing of music as a technology of self is vital to the affordances that musical activities offer as bridges back to “normal” cultural participation and to the ways in which an ill person’s identity exceeds his or her illness. DeNora [9] argues how music’s meanings and effects are constructed, or how they are appropriated, or how patterns of appropriation are manifested. Music’s force is made manifest through appropriation and reception. Within these constraints, however, it is perfectly reasonable to speak of music as a material of social organization, because styles of movement, and emotional and social roles come to be associated with it and may issue from it. 

Music may indeed be conceptualized as a prospective device of agency, and a way of cueing or tuning in to the ongoing formation of order, or, more accurately, “pools” of order, locally achieved. According to DeNora [9,80], music’s effects come from the ways in which individuals orientate to it, how they interpret it, and how they place it within their personal musical maps which link the effects to extra-musical associations. Music is a resource for identity construction and provides a “ground of being”. From this viewpoint, music’s role as a resource for configuring emotional and embodied agency is not one that can be predetermined.

Another source of inspiration is Sloboda’s [81] empirical study of emotional response to music, illustrating how music is capable of arousing deep and significant emotion in those who interact with it. Sloboda showed that individuals with a lifelong commitment to music were much more likely to report strong emotional reactions to musical content than those individuals who were not involved with music [25]. Thus, it seems that musicians who have had many strong emotional reactions to music may be better equipped to mobilize knowledge of the emotional consequences of expression in their performance. Viewed from this perspective, music is the reason why many people engage with music, as performers or listeners, since it has power to evoke or enhance valued emotional states [24]. 

Sloboda and O’Neill [82] showed how music listening in everyday life seemed to make participants “feel better”. Music is a cultural material (as in language) that provides a kind of semiotic and affective “power” which individuals use in the social construction of emotional feeling and displays. As such, the impact of music on emotion is not direct but interdependent on the situation in which it is heard. Any meaningful account of music’s role in the emotional response of individuals must involve the recognition of these complex social factors. The unique capacity of music to engender emotional release or to help individuals break out of anxiety, or for achieving particular emotional states often includes “peak events” or experiences in the stories of their developing commitment to music. This notion seems to relate music to personal life experiences; thus, music is individualistic and cannot be systemized, which suggests that music serves a major psychological function in many people’s lives, closely connected to the explicit purposes of many forms of psychotherapy [24]. According to Sloboda, people look to different pieces of music at different times of life and in different situations because they contain material relevant to particular types of re-appraisal. 

Following these lines of argument, musicking [79] is an often overlooked, but extremely powerful medium of world-making, and an important component of our understanding of ourselves and our relationship with other people. The term “to music” is not concerned with valuation, but seems to be descriptive. It covers all participation in a musical performance, whether it takes place actively or passively, whether we like the way it happens or whether we do not, whether we consider it interesting or boring, constructive or destructive, sympathetic or antipathetic. From this viewpoint, music does not express the “essential” qualities of any group or individual, but is formed and changed in the constant process through which people sustain, modify, transform, and abandon conventions. In other words, music participates in the construction of meaning by building social worlds, identities, bodies, and situations [83,84]. This theme lends itself to empirical investigation, to the extent that musical world-making practices and their consequences can be tracked and documented. When someone says of a piece, “It is not as good as” or “what I really like is”, they are making an evaluation that draws on simultaneous recognition of other texts, experiences, or performances [85], such that these experiences, worlds, and stories may reward further exploration of how music can be a vehicle in gaining a way of knowing [83] or ontological security [86].

A stimulus-response model of how music works eludes the meaningful, interpretive acts of music recipients as they draw upon music’s “affordances” as part of mundane musical practice [9]. Similarly, studies of the musical composition of the body, therefore, complement ongoing discussion and debate in the sociology of health and illness as they address the so-called “mind/body” issue [87]. In short, extra-musical, contextual matters need to be taken into account; for example, a sociological understanding of music may be approached through an examination of musical activities as collaborative interactions [23].

### 1.3. Gaps in Knowledge

Music-based interventions seem to be inconsistent, and practitioners vary in their professional training and preparation for implementing music-based clinical strategies [60]. Research on quality in nursing care facilitators shows that, despite competence-enhancing measures, they are still facing resistance to change and difficulties in the implementation of innovative ideas [60,88]. Research continually produces new findings that can contribute to effective and efficient healthcare [25]. However, such research cannot change outcomes unless health services and healthcare professionals adopt the findings into practice [89]. Systematic development programs in elderly care are required to improve staff skills. Staff attitudes are key challenges to success [90]. 

As described by Lundvall et al. [91], an implementation phase involving many actors in problem solving needs to be an open communicative process, so as to avoid complications and barriers like gatekeeping. Research pointed to how implementation of new technologies in healthcare settings may upset established roles, procedures, and cooperative relationships, as well as create uncertainty and insecurity in everyday work, beyond the psycho-social working environment [29]. These aspects need further investigation in reference to music-based environmental interventions. 

There is a call for further research of how music-based environmental interventions are implemented in daily life in nursing homes, and evidence of how it works in practice. To ensure a sustainable care service in the future, there is a need for a professional change that requires higher levels of competence and new professional approaches [92], which also includes music-based interventions [61]. Changes in practice in nursing homes can be challenging [90], and it is essential to get all of the staff involved in implementing new methods [93]; hence, a stronger focus on the various aspects of the musical impact in elderly care is called for. The inclusion criteria and definitions for music therapy and music-based interventions vary greatly, making transfer to clinical practice even more challenging. In addition, specific interventions being offered are generally described with little detail [60,94].

### 1.4. Purpose and Aim

The main purpose of this paper was to increase understanding and knowledge of how and why the systematic use of musical intervention can be an integral part of nursing education and its implementation in healthcare service. 

The aim was to explore, describe, and identify experiences, expectations, attitudes, and beliefs among nursing students, practice counselors, teachers, and project leaders in reference to the implementation of environmental music intervention, using the “Myskja model” at three nursing homes and healthcare centers in Norway. 

## 2. Materials and Methods

### 2.1. A Qualitative, Cross-Sectional Case Study

Qualitative methods are increasingly recognized in medical and public health research [95,96]. A thorough analysis, leading to reflexive stories that can make a difference, distinguishes a scientific approach from superficial conjectures [96]. In qualitative analysis, knowledge is developed from experiences by interpreting and summarizing the organized empirical data. This study is a cross-sectional study from a region in Norway (2017) and had a qualitative and explorative approach. A qualitative method approach is well suited to gain insight into the participants’ experiences, thoughts, and feelings. It enables researchers to answer how and why type questions, while taking into consideration how a phenomenon is influenced by the context within which it is situated [96,97,98,99]. Since this study employed a qualitative approach, theories and a conceptual framework are only presented to underpin knowledge in the current field. We tried highlighting our results and major findings in relation to previous research and theories that seemed appropriate.

### 2.2. Data Collection and Analysis

The sample, aged from 20–64 (*n* = 33), was strategically and conveniently selected, and consisted of two groups of nursing students (*n* = 15), two groups of counselors (*n* = 9), three supervisors (*n* = 3), one project group (*n* = 4), and two leaders from the nursing homes (*n* = 2). 

Passive observations lasting two days in each of the six departments were executed in order to observe the musical environmental intervention in practice. These visits took place in the same period as the focus group interviews.

Seven focus group interviews were conducted during the spring of 2017. A single interview guideline was designed and used by both researchers. It was an open-ended guide which gave both structure and flexibility to enable themes to emerge from the informants’ own accounts. The focus group interviews varied from three to eight participants, and lasted for two hours on average. The interaction among the participants stimulated discussion to complement, challenge, and suggest alternative ideas. Since this was a cross-sectional study, participants were not followed up over time. Digital audio recordings of all interviews were transcribed between interviews, enabling the process of analysis to begin and to influence subsequent data collection [97,98,99,100]. It is vital to point out that there were no pre-defined categories, concepts, or themes to organize the data. To label the material was a highly personal and individual task completed by the researcher. In general, researchers might name or label some of the data differently; their findings, however, should not differ given the same dataset. The analysis was a synthesis of systematic text condensation (STC) inspired by Malterud [96] and elements from grounded theory by Charmaz [100,101]. The potential of grounded theory methodology lies in the fact that it gives voice to the actors themselves, enabling them to construct their realities through action and process. Grounded theory permits conceptual knowledge to be derived through close association with the data. It can enrich our understanding of music’s mechanisms of operation in naturalistic settings in ways that are derived from the meaning given by systems of people (respondents) themselves.

Systematic text condensation (STC) is a descriptive and explorative method for thematic cross-case analysis of different types of qualitative data, such as interview studies, focus groups, observational studies, and analysis of written texts. The advantage of STC is that its applicability is not restricted to specific types of empirical data. 

STC is a strategy for analysis developed from traditions shared by most of the methods for the analysis of qualitative data. However, the method offers researchers a process of intersubjectivity, reflexivity, and feasibility, while maintaining a responsible level of methodological rigor. If other researchers are given the transcripts, and basically the same analysis, they can follow the procedure and progress, and validate the conclusions. However, the role of the researcher needs to be taken into account (see Section 4).

The method was a four-stage pragmatic approach inspired by phenomenological ideas [96]. It started with a holistic view of the data, and then divided the data up into meaningful units with codes and sub-topics. The third step of analysis implied a systematic abstraction of meaningful units within each of the code groups established in the second step of analysis. In the fourth step of analysis, data were re-conceptualized, putting the pieces together again and synthesizing from condensation to descriptions and concepts. 

Identifying themes were linked with significant units, such as text containing opinion knowledge. The topics related to the interview guide and issues, and were added to analytical text accompanying the various categories. We developed knowledge from the participants’ experiences by interpreting and summarizing the organized empirical data. 

In addition, inspired by grounded theory, the research questions attempted to explore and describe the impact of social processes on human factors in the implementation and adoption of innovations in healthcare as they emerged from the ethnographic data [101,102]. The inductive nature of grounded theory methods assumes an open, flexible approach, shaping methodological strategies while engaged in the research, rather than having them planned before beginning the data collection. This notion of flexibility is important to let themes emerge from the informants’ own accounts. Recurrent themes can be described as themes that occur several times within one participant and/or among the sample as a whole. The first major analytic phase of the research consisted of coding the data with open coding in order to identify descriptions of thoughts and ideas related to the interview questions. To generate categories and subcategories, focused coding was used to compare between incidents, contexts, and situations, and connections between incidents, situation, and categories were explored.

### 2.3. Planning the Music-Based Environmental Intervention—The “Myskja Model”

It might be well worth mentioning that music-based intervention here is not the same as professional music therapy in this context. There are several music-based interventions, and the “Myskja model” [102] was used as a background for the collaborative project. However, the music-based intervention is called “therapy” in the Myskja model, but this labeling is not discussed here in this paper. In this study, we decided to call it music as an environmental intervention or sometimes a music-based intervention. This environmental music intervention was based on the research, perspectives, and teaching of Myskja, a medical doctor with a Doctor of Philosophy (PhD) in integrated music in nursing homes. The aim of this paper was not to evaluate the “Myskja model”. There are several models and programs that focus on music as an environmental intervention in elderly care. The Myskja model is only one of many. For further information, see Myskja’s publications [62]. His aim was to develop future environmental song and music “therapy” for dementia sufferers. Myskja spent 15 years developing this approach through research at approximately 20 institutions in six of Norway’s counties. Results show that music therapy reduces complaints in patients and reduces their need for medication. Myskja also published books and papers on this theme [103,104]. The trust’s support will help document the project’s methods and to spread the “Myskja model”. The trust supports research on dementia since 2003, including work pursued at Kavli’s (The Kavli Trust, Norway: https://kavlifondet.no/en/kavli-trust-programme-health-research/) research center for geriatrics and dementia.

Nursing students were introduced to the “Myskja model” with a two-hour lecture at their Nursing Education Department and “some” tuition from supervisors and practice counselors. The lecture was held by teachers and nurses from the health centers and nursing homes who had taken a “course” at the “Myskja school”. This project involved efforts to invoke awareness of the various ways in which music could be used in daily care. The claim was founded on the perception that nursing students need encouragement to initiate music-based interventions when they enter into practice. The aims for music-based intervention by Myskja that were presented at the onset for the nursing students and supervisors are described below. 

Nursing student:

- Acquire knowledge of different mapping tools that are used to systematize individual customized music therapy. This may, for example, be an assessment of music preferences.
- The act of mapping involves (i) mapping the patient’s relationship with music; (ii) using prerecorded compact discs (CDs) which contain snippets (mostly 50% of each track) of different musical pieces within different genres; (iii) mapping music preferences using a standardized form; (iv) setting up individual music programs (what to listen to) or group-based music programs (songs or participating in singalongs or attending concerts); (v) developing measurement/evaluation (tools); (vi) documenting (filling in a form or writing a log to be inserted in the patients’ individual care plans).
- Gain experience in gathering up-to-date knowledge, and assessing and communicating this knowledge.
- Participate in/take responsibility for designing a method of measurement related to music therapy, in the latter part of the practice period.
- Attempt to document and evaluate the effect/non-effect of this treatment, as well as make an assessment for further follow-up.
- Examine the relationship between systematic music therapy and the clinical assessment process.

The supervisor:

- Acquire knowledge of different mapping tools that are used to systematize individual customized music therapy. This may, for example, be an assessment of music preferences.
- Further develop expertise in music therapy and planning, and mapping selected patients, ongoing evaluations, and follow-ups.
- Further develop the competences of assessing, facilitating, and communicating current knowledge from various sources back to daily practice.
- Try to establish and use electronic patient records.
- Further develop competence by supporting the student in training on assessing and communicating knowledge in connection with music therapy.

For both nursing students and supervisors, the information from the mapping of musical preferences was to be documented in the individual action plan. The action plan description contained what to do, and this was the start of the practice period. They had to initiate the measure, and conduct music therapy with the selected patient (this was documented during the report). This happened in the middle of the practice period, and the results were evaluated in a report at the end of the practice period.

### 2.4. Ethics

All participants were given written information about the project prior to data collection. All participants had to sign written consent. It was emphasized that participation was voluntary, with the right to also withdraw from participation during the study. They willingly consented to participate, and there were no problems of access to the field of study. The interviews were recorded and transcribed verbatim. NSD. This project was declared not to be subject to notification in February 2017 by The Data Protection Official for Research, Norway (http://www.nsd.uib.no/personvernombud/en/notify/index.html), since all electronic data processed through the entire research process were anonymous. In addition, no sensitive data could be linked to directly identifiable personal data or indirectly through a combination of background information, such as place of residence or institutional affiliation, combined with data on age, gender, occupation, or university. In carrying out interviews, no personal data were recorded, only exclusively in the form of notes. These notes, including recordings, were maculated to ensure that no names and no personally identifiable background information were registered in the data material. Respecting the needs and wants of the participants was paramount throughout the study. An issue with small studies is often protecting confidentiality, as people may be identified by their experiences or expressions; thus, we were sensitive to this. The participants are represented though fictitious names. Consent forms were kept securely and separately from transcribed data.

## 3. Results

### 3.1. From a Period of Confusion to Increasing Awareness of Music’s Power

Students and staff at several departments felt that they gained insight and competence in music-based environmental intervention as part of the interdisciplinary collaboration. They also learned that this required planning, ongoing evaluation, and follow-up. The songs and the music played for the patients illuminated interaction, communication, and development of caring skills and created increased interest and commitment among some of the supervisors and teachers.

The nursing students were interested, and completed and documented with very good results. A student took action with a patient with long-suffering dementia and then said that this was their best experience throughout the practice period, and the experience was very positive.

(Veronica, supervisor)

The nursing students previously had minimal knowledge, insight, and competence on music as a health resource or as a health-promoting activity. An introductory course (two-hour lecture) held at the Nursing Education Department motivated them to search for more literature, and inspired them to put what they learned into practice. Some students even sought out literature on music therapy and music and health that was not part of their curriculum. Several students also previously used singing and listening to music when they had temporary employment in elderly care. The majority of the students said they “heard” this was useful and could create a good atmosphere in the nursing homes. However, they had little theoretical knowledge of these issues from before. As a result, the beneficial effects of using music as environmental “therapy” or “treatment” increased among the students as they gradually experienced “how it worked” in everyday practice. As Mary said,

“It’s amazing to see how songs could work as painkillers”.

(student)

Several of the students reported how they could “observe” and “see” how music “worked”. The patients seemed happier and more content, smiled and laughed more, and were easier to communicate with when music was a connecter and a bridge builder. Some of the students sang songs while they executed their daily care. If the nursing students sang simple folksongs, some patients found relief and comfort. The nursing students could often use singing in situations which could be difficult to handle, for example, when waking them in the morning, or getting them out of bed or into bed. In some situations, singing or listening to music also helped change the mood of some patients that struggled with anxiety, depression, and loneliness. Several students noticed how music could replace medicine, due to its calming and soothing effect. Anna illustrates this with the following quote:

When you see it can work, it makes everyday life easier for you and the patient. Often, instead of medicines, I mean, sometimes we wanted to try music instead of medicines.

(student)

Students, who participated actively in musical interaction such as improvisation, singing, and music-listening with the patients, were engaged, committed, and motivated, as opposed to varied enthusiasm from staff and management. Improvisation here meant that they often could sing lullabies in the evening for them observing its calming effect, or putting on CDs when they observed patients were sitting alone or were passive for a long time. These elements are illustrated through Camilla’s narrative.

Yes. For her, we found how it works best. She [the patient] increased her quality of life by appreciating listening to music… when we were making a playlist for her, she almost started crying because she was so happy that she should have a playlist with music that she could hear when she wanted to… so it may change your mood, I noticed it; special songs give totally different feelings.

(student)

The students learned that they can use music and singing in many contexts. Music seemed to provide vitality, joy, and sense of belonging to a specific context or situation. The students particularly noticed how music listening resulted in calmer and more satisfied patients, even after the music or singing event ended. Sometimes, they also started narrating stories for the students. In other words, through music listening or singalongs where patients often recalled memories, the contentment and relaxation seemed to sustain for a longer period of time. In other situations, some students also sang for their patients and, at times, they sang together.

“It was one morning, I remember, when she [the patient] was very upset… then, I sat down and sang with her… and then she was at ease for a long time. I saw this quite often”.

(Mona, student)

In this sense, the students became aware of that music has an effect, and how it also had a positive impact on the environment in the department, improving working life. The nursing students reported how music and singing songs for the patients also had an impact on their own reflections and consciousness about music’s power to construct wellbeing.

I am pleasantly surprised, I had no expectations, I never thought I would like to enjoy my job so much, I never thought I would find such a wellbeing, but I have found that.

(Lisa, student)

Together with a need for a song, or a musical listening event, one of the nursing students recalled a special event which transferred a patient from boredom to feelings of joy, vitality, and energy.

My patient is 90 years old, struggling with boredom in the evening and sitting and pushing the clock all the time; so, I thought we had to find something to do, so we went through the CDs and it was very positive and suddenly she just lived… and she started singing, jazz, and dancing, and then there was a completely different side of the patient suddenly, and, now, relatives are going to fix the CD player for her, she is very fond of typically Norwegian music as long as the songs are not too slow.

(Freddy, student)

At the nursing homes, they sometimes held different arrangements, like concerts, sing-alongs, and dancing festivals, which seemed, at times, to revitalize some of the patients from being “normally” passive to actively taking part in a musical event.

We arranged a dance festival event lately. Then, they came from the whole department. Everyone seemed to enjoy themselves very much, but it was one thing I noticed. One who has Parkinson’s and has trouble just getting out of bed, he danced swing with me three songs in stretch! And it’s solely because of music, dancing Mazurka.

(Alfred, student)

### 3.2. Barriers and Success Factors on Implementing the Music-Based Intervention

Several students, teachers, and supervisors experienced barriers and resistance in implementing the music-based environmental intervention among leaders, staff, and management. Two supervisors admitted they were unsure of the practical work with the music intervention and they came to realize that the students also lacked specific knowledge of procedures.

We thought the students would guide us and they thought we would guide them.

(Sarah, supervisor)

Lack of information to what they were supposed to do in practice and in concrete, practical situations with the music was a frequent challenge. There was uncertainty about what should be done, when it should be done, and who should attend from the various departments. It may seem that, in some situations, the theoretical knowledge base was not quite sufficient for the students and supervisors. The implementation process did encounter obstacles and hindrances on the way, and the systematic environmental music intervention turned out to be more randomly executed. 

The quote, “there is no way in which management at these departments facilitates participation in training and implementation of music and singing in practice” (supervisor), illustrates some of these barriers. Several members of staff and management did not attend the information meetings that were organized by the teachers from the Nursing Education Department. The implementation process itself was a challenge for both students and supervisors. Knowledge of music as an environmental “intervention”, “treatment”, or “therapy” was often mixed by most of the participants, which did not help the situation when they were confused about what they were actually going to achieve or “do”.

I had supervisors who did not at all know what to do. I did not get help. I’ve used music for patients who had a lot of uneasiness and anxiety. A patient said that she would go home and visit her mother-in-law and, when we locked the door, she was sad and crying. Then, I used music to calm her and to reduce her confusion. We used her CDs. I had little information about what kind of music she liked, but I read in the journal and filled in the form what she said she liked.

(Emma, student)

The act of mapping the patients’ musical preferences, likes, and dislikes, often resulted in some frustration. Due to lack of knowledge of the mapping process, and a solid theoretical background of music’s role, they seemed to have little awareness of the patients’ reactions and behaviors. Consequently, the nursing students experienced the mapping of musical preferences as a relatively time-consuming affair. The students admitted that they were not specifically trained or prepared for the music-based intervention, despite their enthusiasm and willingness to work with this in practice.

We have not really learned so much, we think it was not really well organized… I think we should have had more teaching, and learned more about it [music] first so we could use it more actively… we were thrown into it. We were actually halfway (in practice), but still had not got any more information since we were at our lecture.

(Fiona, student)

Several of the students, despite their motivation for using music as a health promoter, explained that they had not really received any special training. As such, it appeared as if the topic in their education was too superficial and not seriously treated. Music and singing are not originally on the curriculum for the nursing students, but since they now were part of this project, they warranted more in-depth teaching of skills and knowledge on this theme. Some students mentioned also that they were not taught how music listening could be both positive and negative for a patient, e.g., raising anger, getting emotional, tearful, or sad.

Yes, it is good that it is health-promoting, but how music also can induce anger or sadness, you have not had a lecture about that, but it can bring memories then.

(Roger, student)

Or, as another student described,

Some songs I personally was very excited about gave completely different reactions [for the patient]; one began to talk about her mum and childhood.

(Grethe, student)

The act of mapping the patients’ musical choices produced a lot of discussion. Sometimes the mapping of musical preferences had to be done in collaboration with family members or relatives, trying to recall pieces of music that seemed to represent their lives, i.e., their memories, associations, sadness, happiness, and longings. Sometimes the pre-recorded CDs from the Myskja model did not initiate any reactions among the patients. In some cases, the students noted how a few patients simply disliked all the tracks on the CDs. The nursing students had difficulty finding any pieces of music to listen to that the patients themselves found attractive or responded to, or seemed to “work”, i.e., when music listening had a positive effect and influence on the patient.

I would have to figure it out in other ways. One patient said “bad music”, but then she said she liked old fashioned music and then I searched for it. She was very certain of what she did not like.

(Hannah, student)

As pointed out, the mapping of their musical preferences did take time and effort, and, quite often, the nursing students used their own songs to be sung instead, or they found their personal playlists from Spotify or YouTube to map their patient’s musical likes and dislikes. As such, the mapping form seemed at times useless.

The mapping form (using the CDs from the Myskja model) did not work well, but the patient had many CDs we used. We all played for a patient twice, and tried to catch and observe different reactions. She [the patient] explained herself that different songs gave different reactions, she realized her emotions changed with the mood of the music, giving different associations… she sat still and listened. Different songs stimulate different reactions… that were amazing. Another patient would not listen to the entire CD, but chose the dance band music we had listened to, and she liked it. She got up and danced with me once, and that person really walks with a Zimmer-frame. I’d never thought I would live to see this.

(Mary, student)

Teachers found that the students’ focus changed gradually from being slightly critical to the mapping process to see the importance of individual mapping that benefited the patient. Mostly, this was due to the nursing students’ own creativity, patience, and motivation to cope with the ongoing difficulties. Quite often, they did what they felt was right in the moment or situation, thinking how they could enhance comfort and quality in the care for their patients. 

Music and singing became a bridge = building act and a connecter of communication between the nursing student and the patient. As such, the act of mapping also resulted in good, enjoyable and inspiring experiences for the nursing students, describing this as memorable and warm moments with their patients.

We were with a patient, who recently moved here, and she had sung in a choir and, through the mapping of her musical choices, we got a good insight into what we could use to prevent uneasiness and turmoil. Once we had gone halfway through the CD and came to rock, country, and dance band, she started telling funny stories, associated and described so well what she thought of and what she dreamt about. Amazing.

(Debbie, student)

### 3.3. Lack of Coordinated Action—The Importance of Management Engagement

It became quite obvious through this data that a key to success needed to start with engagement, motivation, and encouragement among those who had responsibilities for the students in practice. For a successful implementation, one needs to have the “entire department; otherwise, it does not work; we have to include all levels”, as several students stated. The nursing students claimed that the supervisors and practice counselors were not always available when they needed advice and help in daily practice. Nurses who attended the “Myskja course” were often away.

It was a bit like this: now we have to start with some music therapy. My supervisor had a lot to do, and regretted that she had not read up on this. Then it’s better to use those who are enthusiastic.

(Anna, student)

Some of the departments had other healthcare personnel who were introduced to music-based environmental “treatment” or “therapy” from the Myskja courses, but were not part of the project. In this respect, if these nurses had more responsibilities for the students, it might have affected the implementation and learning process in a positive direction, as one student claimed. 

If it had it not been for them, well, I am not sure if we did manage to do this music-thing.

It was also pointed out how mood, prejudices, and attitudes among the management influenced the students in their daily practice. The staff did not always encourage or help the nursing students in their “musical tasks”. It was pointed out by some of the students that nursing and caring did not involve music and singing. It was not part of the educational program either and, consequently, not something the staff or management needed to take seriously or put on the agenda.

Yes, it’s a problem that not everyone is as keen.

(Susan, counselor)

This was illustrated in the following quote:

When someone is negative it’s an infectious mood; what’s also strange is that when those who are negative performed music “under compulsion”, it never worked.

(Heidi, counselor)

Since music-based interventions are not part of the nursing education here, the students asked for this topic and theme to be part of their education, due to their acquired curiosity and raised consciousness during practice. An explanation of why they had numerous difficulties both at the onset of the project and during the period in practice seemed to be a lack of coordination and cooperation from actors involved. Statements from the participants that were involved in the project team underlined the significance of including music-based intervention as part of their aims and action plan at the nursing homes.

If we had used the local Myskja contacts more, this would have been much better; but we were not aware of this in advance, so the entire department could follow up and that would be positive. I think we have kindergarten cooperation and the students had been very motivated and thought this was very rewarding. We have not tried this [music-based intervention] systematically before the students arrived. It is not systematized and added to our aims really, and in our action plan, I do not know the reason why the Myskja contacts did not work more with implementation in advance. There are still some who work with us who do not know what this is.

(Alice, leader, nursing home)

The project leader participated only in the first supervisory meeting, and there were none from the project team employed in the department who could have a significant influence on the implementation process or motivate and encourage the students in their practice. Sometimes, it was even difficult to encourage patients to participate in the music procedures, such as listening to music or participate in sing rounds in the sitting room. These aspects led to a heightened frustration among the nursing students who were originally enthusiastic and motivated about the music-based intervention. Lack of coordinated action, and solid knowledge and information about the music-based intervention among a few actors involved seemed to increase the nursing students’ insecurity and uncertainty of what they should do in specific situations and events.

I think they (management) should have known a little better in advance. What I experience as a problem was that we did not get enough information about what to do. It would be better if we had a clear message of how it should be done. We experienced that our supervisors knew as little as us. We had someone else at the department [not in this project] who had a book we borrowed. We should take some tests beforehand; this should be done by the supervisors beforehand, but it did not happen.

(Lisa, student)

## 4. Discussion

### 4.1. Strengths and Limitations of the Study

In qualitative studies, it is important to reflect upon and consider the role of the researcher(s). The “observer effect”, i.e., the researcher’s gender, age, and personal characteristics, needs to be taken into account [98,99]. However, a researcher is not without history, and needs to be reflexive throughout the whole research process. In this respect, our personal “style”, enthusiasm, and interest in doing the fieldwork may have influenced the participants in a positive manner. An aspect worth considering is whether these results would have been comparable if someone else conducted the research. At the same time, there was an-ongoing process of self-reflection during this project in our role as researchers, which we hope contributed to the trustworthiness and credibility of this study [95,96,97,98,99,100,101]. 

Another feature of the study is the fact that it may be difficult to elicit data about a complex topic in one-off interviews at one point in time and, hence, fewer possibilities to establish a “deep” rapport or thick descriptions [105]. Our data are primarily composed of one-off in-depth focus-group interviews. Moreover, as data were collected and analyzed, we were both involved in the process of checking our interpretations. In this respect, we had the opportunity to discuss and clarify the interpretation, and seemingly contributed to additional perspectives on the issue under study. The interaction in the focus group may uncover tacit knowledge and experience-based knowledge from the field, giving an awareness-raising effect on the participants. In this sense, they could compare their own experiences with others and, thus, identify factors that seemed relevant to the research topic [99].

Since this was a cross-sectional case study, it did not have the advantages of a longitudinal study, which enables a comparison of participants’ experiences and compares one point in time with another point in time, thereby linking findings to context, situation, and meaning. Comparing processes and changes to how participants relate to the collaboration and interactive process could have revealed how practices, expectations, and attitudes would have changed over time [90]. An action research approach would have also possibly given more solid and rich data to the processes and changes during the project [106].

Regarding the one-to-one relationship between interviewers and informants, the individual’s experiences in qualitative interviews are a dialogue, which formulates the interviewee’s own life world. Group interviews also provide this type of information; however, through the participants’ shared experiences, both among themselves and with us, the group may exert a pressure that inhibits individuals from speaking freely. Through individual interviews, we could have probably collected more knowledge of individuals’ unique experience and understanding of this phenomenon. Despite this, the group may have stimulated each other on topics and issues not necessarily explored in the individual interviews. 

### 4.2. Increasing Awareness of Music’s Power

As we noted, students and staff at several departments felt that they gained insight and competence in music-based environmental intervention as part of an interdisciplinary collaboration. They also learned that this required planning, ongoing evaluation, and follow-up. The nursing students previously had minimal knowledge, insight, and competence with regards to music as an environmental intervention, as a health resource, or as a health-promoting activity.

The songs and the music played for the patients developed caring skills and created increased interest and commitment among the nursing students, particularly when they could see and observe how it “worked”. Through musical engagement, the communication among the nurses and the patients was enhanced, producing joy and awareness of music’s power as a health resource and provider of vitality [2,3,4,5,6,7,9,13,20,25,26,29,30,40,55,56,68,80]. 

A stronger focus on theories related to music as ways of providing meaning and sense of coherence in life [6,7,77,78] and empowering our lives could perhaps create a better understanding of music as a health resource. These results highlight how music may provide resources for reminiscence, and for the adoption of roles and emotions in, during, and after musical activity [42]. According to DeNora [7,8,80], music’s effects come from the ways in which individuals orientate to it, how they interpret it, and how they place it within their personal musical maps which link the effects to extra-musical associations. These data also show how music-based interventions can be an alternative to medicine, or a supplement that reduces the need for it [1,21,22,29,60,107]. To be certain, alternatives to medication should be investigated, in line with a new focus on non-pharmacological approaches [1,60,102,107].

### 4.3. Barriers and Success Factors—A Need for Further Knowledge and Training

Grudinschi et al. [108] examined challenges in the management of cross-actor collaboration in elderly care in Finland, finding how challenges related to decision-making were mainly at the higher level of management. In this respect, these challenges regarding actors’ strategic ability to create social value in cross-actor collaboration [109] also appeared in this study. Previous research also claimed that feelings of being left out or left behind are common in both private and public actors [110,111]. If we view this music-based intervention as an innovation process that unfolds over time, it might be troublesome and reiterative, and might involve two steps forward for one step backward plus several side steps [112,113]. 

Both nursing students and their supervisors experienced the mapping of musical preferences as a relatively time-consuming affair which they were not specifically trained or prepared for as professional music therapist are. As such, this study highlights how important particular choices of music are connected to patients’ life stories. Pre-recorded snippets of music as illustrated through the Myskja model do not always work, as shown in this study. As authors [5,114,115] argue, the unique set of an individual’s personal experiences may influence which locations, categories, associations, reflections, and evaluations that individual draws upon in interpreting music as personally relevant. 

More in-depth focus on theoretical knowledge and training ought to be taught to the nursing students. For example, previous research underpinned how the mapping of personal musical preferences is important for patients outcome, and how the flexibility of such methods is a vital feature. Identifying the participants and the mediators would make the process to create a track list on the CDs personal and individual each time. In other words, there is no standard designed music to be given from the mediator to the participants; rather, personal music is given to the mediator or facilitator (researcher) from the participants [1,5,29]. In this study, snippets of prerecorded music were mainly used to tap into the musical preferences of the patients. A challenge and issue here is how nurses and nursing educators can or ought to develop their competences without the use of professional music therapists in situ or someone with similar professional competence. 

If nurses are to be trained in musical intervention, some scholars also suggested that nurses ought to develop their personal healing qualities which might increase awareness of healing in their own lives [44,45] and that, thus, the time has come to make music intervention an integral part of nursing education practice [5,51,94,102]. 

### 4.4. The Significance of Interdisciplinary Collaboration—The Value of Engagement

Interdisciplinary collaboration is an essential element of any environmental intervention and innovation [94,108,109,110,111,112,113]. A recent study of explored resistance to the implementation of welfare technology in municipal healthcare services [116] highlighted issues like threats to stability and predictability or fear of change, threats to role and group identity, fear of losing power or control, and threats to basic healthcare values. Our research supports these findings in terms of problems, challenges, and barriers with the initial phases of adopting the music-based intervention among the leaders and management. To prevent further resistance in complex co-creation processes [116] and to reduce levels of frustration and criticism of co-actors (here, nursing students), a heightened level of motivation and knowledge for music as a health resource is needed both among the management and staff in general. 

As we saw in this study, commitment for music among employees and management seemed to vary with the degree of motivation and attitudes toward music-based environmental intervention. Employees also seemed to lack knowledge, competence, and skills on the systematic use of music in elderly care. As music is a ubiquitous medium and very much embedded in everyday life [9], once people are encouraged to learn how to use it as health resource, it is the most pragmatic, cost-effective, and sustainable means that can be utilized even after the actual intervention period ends [22,29]. If the systematic use of music in elderly care can be successful, it seems vital to include this theme in nursing education. By creating early involvement among nurses, it might also influence, inspire, and encourage involvement among employees and management to use music as an environmental intervention [63,64], as a way to strengthen co-creating processes and interdisciplinary collaboration [88].

By building strong ties between the educational system and health institutions, both cultures might build stronger bridges and connections to increase coordinated action, planning, and implementation. These learning processes also demonstrate how innovations grow, are nurtured, and meet problems, and how some of them fail; thus, van de Ven et al. [113] call this the innovation journey. This case study highlights how the majority of the participants may have increased their awareness of music’s power, and consciousness of the fact that they seemed to acknowledge how to solve future challenges and reduce failures, which itself is a phenomenon of significance to recognize [117].

## 5. Conclusions

Increased knowledge and insight into various types of musical, methodological approaches, teaching methods, and mapping of musical preferences seems to be necessary in order to provide more thorough and broader training for nurses during practice. By facilitating opportunities for good learning, the nursing students may use singing and music as part of their art of caring, enhancing environmental care for the elderly. As previous research from several disciplines also shows, music has good effects for patients, staff, and relatives, which often benefits the psycho-social working climate. Since this study only involved one music-based intervention, other musical procedures and approaches could be considered for future research and practice. 

To summarize and conclude, there are six themes to consider for future research and practice.

- To acknowledge the unique capacity of music for achieving particular emotional states, the chosen music seems to be related to personal life experiences, contexts, and life phases. Thus, the benefits of self-selected music are a vital feature in music-based interventions.
- The act of mapping musical preferences is vital, but could perhaps be guided by professional music therapists or someone with similar competence to avoid barriers and to aid the implementation and adoption process for all actors.
- If nurses aim to learn how to incorporate music intervention into their practice, theoretical and evidence-based knowledge must be taught by teachers and similar professionals from educational institutions.
- To achieve success and lessen barriers in the implementation of music-based interventions in nursing homes, there needs to be a raised level of commitment, and changes in attitudes and beliefs in what nursing may contain and involve. A solid backing and the use of a professional music therapist or likewise to support and aid this process would possibly contribute to better coordination and heighten the level of engagement and motivation among actors involved for better quality of care.
- To introduce new approaches and methods in healthcare practice is challenging. If management could appreciate how music and singing may enhance a health-promoting workplace for its employees, it might be possible to create a stimulating, rewarding, and thriving psycho-social environment, which would benefit all actors involved.
- Music and “musicking” as a method or strategy in elderly care ought to be included as a vital component for the enhancement of health, wellbeing, and quality of life for patients in nursing homes. This is in line with geriatric care, which recommends non-pharmacological approaches as a complement to conventional care.

Taking all issues into account, somatic and medical care has come to dominate public healthcare, while interaction and relational care practices are more marginalized; hence, music-based interventions ought to be systematically introduced into healthcare settings as a sustainable and health-promoting activity in the future.
